# The Zebrafish Insertion Collection (ZInC): a web based, searchable collection of zebrafish mutations generated by DNA insertion

**DOI:** 10.1093/nar/gks946

**Published:** 2012-11-23

**Authors:** Gaurav K. Varshney, Haigen Huang, Suiyuan Zhang, Jing Lu, Derek E. Gildea, Zhongan Yang, Tyra G. Wolfsberg, Shuo Lin, Shawn M. Burgess

**Affiliations:** ^1^Genome Technology Branch, National Human Genome Research Institute, National Institutes of Health, Bethesda, MD 20892, USA and ^2^Department of Molecular, Cell, and Developmental Biology, University of California, Los Angeles (UCLA), Los Angeles, USA

## Abstract

ZInC (Zebrafish Insertional Collection, http://research.nhgri.nih.gov/ZInC/) is a web-searchable interface of insertional mutants in zebrafish. Over the last two decades, the zebrafish has become a popular model organism for studying vertebrate development as well as for modeling human diseases. To facilitate such studies, we are generating a genome-wide knockout resource that targets every zebrafish protein-coding gene. All mutant fish are freely available to the scientific community through the Zebrafish International Resource Center (ZIRC). To assist researchers in finding mutant and insertion information, we developed a comprehensive database with a web front-end, the ZInC. It can be queried using multiple types of input such as ZFIN (Zebrafish Information Network) IDs, UniGene accession numbers and gene symbols from zebrafish, human and mouse. In the future, ZInC may include data from other insertional mutation projects as well. ZInC cross-references all integration data with the ZFIN (http://zfin.org/).

## INTRODUCTION

Functional genomic studies in different model organisms including vertebrates such as mice, frogs and fish have contributed immensely to our understanding of various human diseases. Furthermore, it is now possible to systematically create gene mutations and then study the associated phenotypes (i.e., ‘reverse’ genetics). Zebrafish has gained momentum in the past two decades as a genetically tractable model organism in which to study vertebrate development because of its transparent embryos, small size, *in vitro* development, high fecundity and inexpensive maintenance (1). Their embryonic development is fast, with most of the critical development occurring in the first 5 days. Traditionally in zebrafish, forward genetic screens are used, and two independent, large-scale mutagenesis screens have been carried out using ENU as the mutagen (2,3). ENU is very efficient, but it introduces single base-pair change into DNA, and the identification of the target gene is typically done by a rather laborious and time-consuming method of positional cloning (4,5). To circumvent the positional cloning step, insertional mutagenesis strategies have been developed in different model organisms. Insertional elements such as transposons and retroviruses have become indispensible tools in manipulating genomes for various applications, not only insertional mutagenesis, but also transgenesis and gene therapy (6,7). Insertional mutagenesis using retroviral vectors has effectively been used to disrupt gene functions in vertebrates (8,9). We are using a high-throughput retroviral mediated mutagenesis followed by mapping using next-generation sequencing methods (10) to generate a knockout library of zebrafish. In order to make this mutagenic resource readily available to the research community, we have developed ZInC, the Zebrafish Integration Collection, an integrated database and web front-end to display mutagenic insertions in a simple and interactive way.

## INSERTION DATA SOURCE

The experimental data in ZInC are derived from our ongoing retroviral mediated insertional mutagenesis project (Varshney and Lu et al., unpublished). A graphical representation of the pipeline is included in the ZInC website.

## INSERTION DATA PROCESSING

We developed a robust mapping analysis pipeline to generate insertion data in the zebrafish genome. Raw data were processed from ELAND or BAM output files, generated from paired-end sequencing using Illumina platform, and sequence reads were extracted. To map insertion sites, retroviral vector and linker sequences were trimmed, then the trimmed reads were mapped to zebrafish genome (Ensembl Zv9.0 assembly) using the short read aligner Bowtie (12). Since LM-PCR was performed to isolate specific integration events, it was possible that the same integration site was sequenced multiple times. Therefore, sequence reads that mapped to the same chromosome, having the same genomic alignment start position, and mapping to the same DNA strand defined a single integration event. The mapped integration sites were then compared to the genomic locations of annotated genes (Ensembl e65) to determine which integration sites are associated with genes, that is, which integration sites are within exons or introns. Insertion events are flagged as being ‘Predicted Mutagenic’ only if they fall within any exon or the first intron.

## DATABASE DESIGN AND IMPLEMENTATION

The ZInC website interface uses a Common Gateway Interface (CGI) constructed in the Perl programming language that interacts with a relational database hosted in Oracle 11g. The web interface was developed by using HTML, Perl, Java Script and Template Toolkit. The connectivity between the CGI and the Oracle 11g Relational Database Management Software was implemented using Perl's Database Interface (DBI) and the Oracle database driver for the DBI module (DBD::Oracle). The ZInC database consists of several tables that hold the data content, including the zebrafish gene name and gene symbols, human and mouse gene orthologs, and zebrafish KEGG (Kyoto Encyclopedia of Genes and Genomes) information (13). The tables are populated by downloading data from DAVID (http://david.abcc.ncifcrf.gov/; KEGG only) (14,15) and Zebrafish Information Network (ZFIN) (http://zfin.org/; all other annotations) (16); these data will be refreshed quarterly, or as available. The list of insertions is also updated as new experimental and bioinformatics analyses become available. A suite of Perl scripts was developed to add new data and annotations into ZInC as well as to ensure data integrity.

## DATABASE NAVIGATION

**Figure 1.**
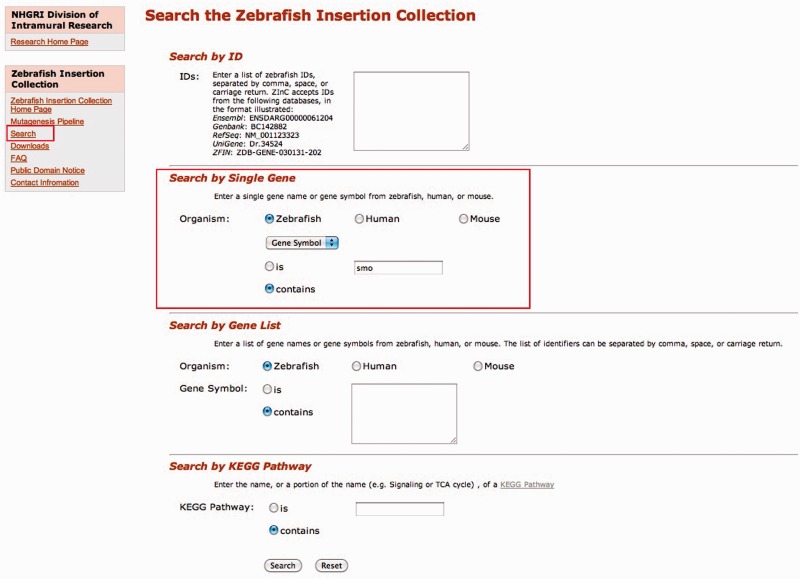
ZInC search interface. Insertion sites within genes have been mapped to a variety of common identifiers. Users can query ZInC with accession numbers from a number of sources, including Ensembl, GenBank, RefSeq and ZFIN. Users can also query by human, mouse or zebrafish gene symbols and names, either individual entries or longer lists. Queries can also be performed on KEGG biological pathways. All searches allow for an exact match (is) or a query with wildcards (contains). In this instance, we searched for a mutant in the gene *smoothened* using the zebrafish symbol ‘smo’ and by choosing the ‘contains’ radio button, the search will return any gene symbol that has the text string ‘smo’ in it.

The navigation sidebar on the left side of each page provides links to different sections of the web resource. As shown in Figure 1, the ‘Search’ link is the main one, a search page that provides access to the integration sites in the database. To facilitate the search, we provide a simple interface that accepts multiple input IDs such as Ensembl (e.g. ENSDARG00000010070), Genbank (e.g. BC081408), RefSeq (e.g. NM_001004678), UniGene (e.g. Dr.134464) or ZFIN (e.g. ZDB-GENE-040912-127) identifiers. The user can enter either a single ID or a list of IDs separated by commas, spaces or carriage returns. Users can also search by gene symbol (e.g. smo) or gene name (e.g. smoothened homolog) from zebrafish, human or mouse. A list of gene names or gene symbols can also be used as search input; again, the list of identifiers can be separated by comma, space or carriage returns. Researchers studying biochemical pathways can search by KEGG pathway (13) terms (e.g. Glycosphingolipid biosynthesis) to find insertions in genes in a specific pathway.

**Figure 2.**
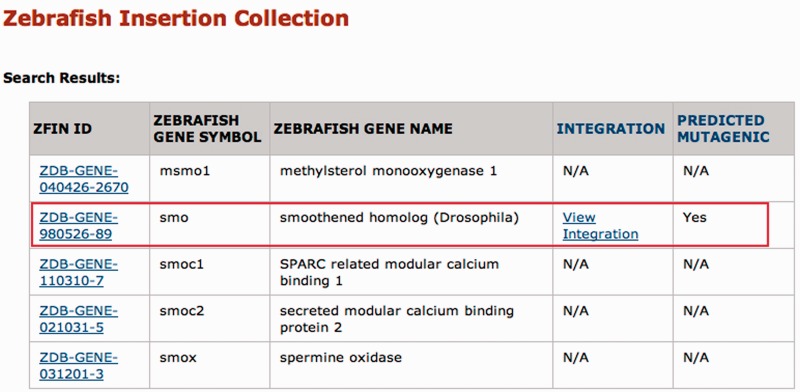
ZInC search results. In Figure 1, the zebrafish gene symbol ‘smo’ was entered in the Search by Single Gene box. Since the ‘contains’ radio button was selected, all zebrafish gene symbols in ZFIN containing the text string ‘*smo*’ are returned, regardless of whether an integration in the gene is available. IDs in the ZFIN ID column link to ZFIN entries for specific genes. For those genes with an integration, more detailed information is available through the ‘View Integration’ link (Figure 3).

**Figure 3.**
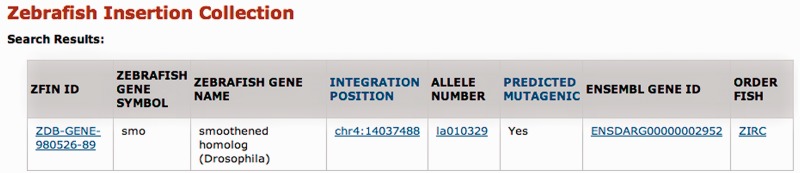
ZInC integration site details. Clicking on the ‘View Integration’ link on a search results page (Figure 2) results in a detailed view of the integration site. The ‘Integration Position’ column links to the UCSC Genome Browser zoomed in to a 2 Kb window around the integration site, the ‘Ensembl Gene ID’ column links to the Ensembl gene page, and the ‘ORDER FISH’ column links directly to the ZIRC to purchase the desired mutant fish (if available).

The results of a search are shown in Figure 2. For each gene hit by the query term, the ZFIN ID, zebrafish gene symbol and zebrafish gene name are returned, regardless of whether that gene is disrupted by an integration. The presence of an integration is indicated by the link ‘View integration’ in the Integration column; whether the insertion is predicted to be mutagenic (i.e. it lands in an exon or first intron) is marked in the final column. The results of the ‘View integration’ link are shown in Figure 3. In brief, this page shows the integration position, allele number, Ensembl gene ID and a link to order fish through the Zebrafish International Resource Center (ZIRC) when available. The insertion position is linked to the UCSC Genome Browser so that users can see the genomic context of each integration site. Each allele number is linked to the corresponding ZFIN allele page.

## CONCLUSIONS

ZInC is a part of an ongoing project where we aim to knock out every protein-coding gene in the zebrafish genome. We will update the database at least quarterly with newly identified integration sites. Other groups are also attempting to knock out genes using different insertional elements, such as the *Tol2* and *Ac/Ds* transposons (17,18). An effort is being made to integrate these and other similar data into ZInC, allowing it to serve as a central repository for all integration sites in zebrafish.

## ACCESSIBILITY

ZInC can be accessed at http://research.nhgri.nih.gov/zinc. Comprehensive lists of all insertion sites, as well as protocols and methods required for the genotyping can be downloaded from http://research.nhgri.nih.gov/zinc/?mode=downloads. All the data cross-references with gene and mutant data in the ZFIN: http://zfin.org.

## ACCESSION NUMBERS

As of August 2012, 13 316 insertion sequences have been submitted to the National Center for Biotechnology Information (NCBI) Genome Survey Sequence (GSS) database. The accession numbers are JS426363-JS426454, JS495495-JS496658, JS578512-JS583384, JS672208-JS672893, JS784708-JS785225 and JS876947-JS886733, and the BioSample ID is GSS: LIBGSS_038780. The full list of integrations is available from the Downloads page at: http://research.nhgri.nih.gov/zinc/?mode=downloads.

## FUNDING

Intramural Research Program of the National Human Genome Research Institute, National Institutes of Health (to S.B. and T.W.) and R01 grant NIH DK084349 (to S.L.). Funding for open access charge: Intramural Research Program of the National Human Genome Research Institute, National Institutes of Health (to S.B. and T.W.) and R01 grant NIH DK084349 (to S.L.).

*Conflict of interest statement*. None declared.
